# From upright to upside-down presentation: A spatio-temporal ERP study of the parametric effect of rotation on face and house processing

**DOI:** 10.1186/1471-2202-10-100

**Published:** 2009-08-19

**Authors:** Boutheina Jemel, Julie Coutya, Caroline Langer, Sylvain Roy

**Affiliations:** 1 Research Laboratory in Neuroscience and Cognitive Electrophysiology, Hôpital Rivière-des-Prairies, 7070 Blv Perras, Montreal, Canada; 2 Centre de Recherche Fernand Seguin, Psychiatry department, Université de Montréal, Canada; 3 Cognitive Neuropsychology Research Group, Université de Montréal, Canada; 4 Concordia University, Montreal, Canada

## Abstract

**Background:**

While there is a general agreement that picture-plane inversion is more detrimental to face processing than to other seemingly complex visual objects, the origin of this effect is still largely debatable. Here, we address the question of whether face inversion reflects a quantitative or a qualitative change in processing mode by investigating the pattern of event-related potential (ERP) response changes with picture plane rotation of face and house pictures. Thorough analyses of topographical (Scalp Current Density maps, SCD) and dipole source modeling were also conducted.

**Results:**

We find that whilst stimulus orientation affected in a similar fashion participants' response latencies to make face and house decisions, only the ERPs in the N170 latency range were modulated by picture plane rotation of faces. The pattern of N170 amplitude and latency enhancement to misrotated faces displayed a curvilinear shape with an almost linear increase for rotations from 0° to 90° and a dip at 112.5° up to 180° rotations. A similar discontinuity function was also described for SCD occipito-temporal and temporal current foci with no topographic distribution changes, suggesting that upright and misrotated faces activated similar brain sources. This was confirmed by dipole source analyses showing the involvement of bilateral sources in the fusiform and middle occipital gyri, the activity of which was differentially affected by face rotation.

**Conclusion:**

Our N170 findings provide support for both the quantitative and qualitative accounts for face rotation effects. Although the qualitative explanation predicted the curvilinear shape of N170 modulations by face misrotations, topographical and source modeling findings suggest that the same brain regions, and thus the same mechanisms, are probably at work when processing upright and rotated faces. Taken collectively, our results indicate that the same processing mechanisms may be involved across the whole range of face orientations, but would operate in a non-linear fashion. Finally, the response tuning of the N170 to rotated faces extends previous reports and further demonstrates that face inversion affects perceptual analyses of faces, which is reflected within the time range of the N170 component.

## Background

It has long been claimed that the effect of inverting faces from their canonical upright orientation constitutes a diagnostic marker for the processing differences between faces and other seemingly complex and monoriented objects [1]. In his seminal paper, Yin [2] showed that while most objects (houses, airplanes, dogs, etc.) are somewhat harder to recognize upside down than right-side up, face recognition is more drastically reduced by stimulus inversion. The disproportionate inversion effect for faces, termed as *face-inversion effect *(FIE), has not only been replicated by numerous behavioral studies [3,4] but has also been linked to spatiotemporal brain mechanisms as revealed by electrophysiological and brain imaging studies (e.g. [5,6]). Nevertheless the putative mechanisms underlying FIE are still a matter of considerable ongoing controversy.

More specifically, two prevailing but diverging hypotheses (i.e., qualitative vs. quantitative) have been proposed to account for performance decrement due to face inversion. The qualitative or dual-process view posits that qualitatively distinct processing modes are used to process upright and inverted faces; a more configural- and holistic-based processing mode being the default system for processing upright faces and a part-based processing mode which is at work when faces are inverted [3,4,7-14]. Under this view, perceptual encoding and memory representation of upright faces rely in some special way on configural (i.e., spatial relations among facial features) and/or holistic information (i.e., in which faces are perceived as an integrated and indecomposable whole), and in a lesser extent on face parts (e.g., isolated features such as eyes, nose, mouth). Numerous behavioral studies have consistently demonstrated that turning faces upside-down dramatically disrupts the processing of configural information while leaving intact local feature processing [9,11]. On the contrary, the quantitative hypothesis suggests that FIE does not cause a shift from one type of processing to another but would rather reflect a quantitative difference in processing facial information, being either configural [1], featural [15-18] or both [19]. For example, Sekuler et al. [18] found that the same discriminative regions, namely the eyes and the eyebrows, are used to process upright and inverted faces. Under the quantitative view, upright and inverted faces are processed in a similar fashion albeit less effectively in the upside-down orientation [1].

One method for considering the question of whether face inversion causes a qualitative or a quantitative change in processing mode has been to investigate the curve of performance decay as faces were gradually rotated from upright to upside-down orientation. Some findings do go some way in favor of the qualitative view, by showing a steeper decay of configural processing by approximately 90° to 120° rotations [20-25]. Studies isolating configural face processing from part-based contributions have shown that while rotation had a linear effect [22] or no effect at all on featural processing [24], configural processing was found to falloff in a curvilinear fashion. For example, in Stürzel and Spillman's study [25], the method of limits was used to determine at which angle of rotation Thatcherized faces lost their grotesque appearance. It was found that the shift in perception from grotesque to non-grotesque occurred somewhere between 97° (i.e., normal to grotesque) and 118° rotations (i.e., grotesque to normal). Similarly, in one series of experiments, Murray et al. [22] found a steeper reduction in perceived bizarreness of thatcherized faces after 90°. This was true for only the thatcherized faces (i.e. spatial-relational distortion), while bizarreness ratings of component distorted faces (i.e. whitened eyes and blackened teeth) increased almost linearly with orientation. In addition, findings from a sequential matching task [24] indicated that while featural changes were detected accurately at all rotations, the number of errors when detecting configural changes differed depending on the angle of rotation, with a peak in errors at intermediate angles of rotation (90°–120°). More recent studies reported a similar range of orientation tuning of configural processing by using either pairs of overlapping transparent faces in upright and misoriented views [26], Mooney faces [21] or aligned and misaligned composite faces [23]. However, other studies do support a quantitative effect of inversion by demonstrating a linear relationship between subjects' performance and rotation [27-30], consistent with the idea that rotation taps on a single and common process. Valentine and Bruce [30] have proposed that mental rotation could be responsible for the systematic detrimental effect of orientation on face processing, as it is the case for several other objects [31]. According to these authors, misoriented faces need to be first prealigned to upright (e.g., via mental rotation) before entry to the face identification system.

In brain-imaging studies using the functional magnetic resonance imaging technique (fMRI), researchers have mainly investigated the activity modulation of the face cortical network induced by face inversion [32]. This included a circumscribed region in the lateral fusiform gyrus known as Fusiform Face area (or FFA, cf. [33]), the superior temporal sulcus (STS, [32]), and the occipital face area (OFA, [34,35]). Reduced levels of activity in the FFA [6,36-38], STS [6,37,39] and OFA [36] have recently been reported for inverted as compared to upright faces [but see 40–43]. More specifically, it was found that among the three face-responsive regions, only the FFA activity modulation by face inversion exhibited a positive correlation with the behavioral FIE [38]. Decreased activity in face-selective regions has been interpreted in terms of failure to engage dedicated mechanisms to process inverted faces, namely holistic and configural processing. Additional activations in regions known to be involved in processing non-face objects (e.g., the lateral occipital complex, LOC) have also been reported in response to upside-down images of faces [40,41,44], a finding that is consistent with the dual-processing/qualitative hypothesis. It has been proposed that the recruitment of additional resources from the object processing system when faces are inverted may reflect a switch in processing strategy, such as a change from a holistic to a part-based processing mode [41,45].

Electrophysiological studies in humans have shown that face inversion affects the latency and/or the amplitude of scalp recorded event-related potentials (ERPs) sensitive to face perception [5,46-55]. However, two debates still prevail about these ERP components. The first debate strives to determine which ERP component is the electrophysiological correlate of face processing and is thus specifically affected by face inversion. Early studies have identified a positive component peaking around 160 to 180 ms over central scalp sites (Vertex Positive Potential, VPP) that was larger in response to faces than to other visual objects and peaked about 10 ms later to upside-down compared to upright faces [53,56]. More recent studies revealed the existence of an occipito-temporal negative potential around 170 ms (N170) that has been linked to the early stages of face encoding. Similar to the VPP, several scalp ERP studies [49,55] have reported a peak latency delay of the N170 to inverted faces, which was often accompanied by an amplitude enhancement [5,47,51,54]. However, some authors showed that face inversion has an earlier onset (around 100 ms) affecting a posterior positive ERP component known as P1 [50,51,57] and its magnetic correlate, M1 [57]. These latter results thus suggest that P1 is probably the earliest ERP component that best reflects configural encoding of faces. However, a recent review of the electrophysiological literature provides strong arguments in favor of the specificity of the N170 FIE [58]. More importantly, a recent study has clearly demonstrated that N170 FIE is functionally tied to the behavioral FIE [59], by showing that the effect of face rotation on N170 correlated significantly with the behavioral rotation effects. No such relationship was found between rotation effects on P1 and behavioral measures.

Furthermore, the second debate concerns the functional significance of face inversion effect on the N170. N170 FIE has been interpreted in different ways. For some authors, amplitude and/or latency enhancement of the N170 reflects the difficulty in processing configural and holistic information when inverting faces [5], and also when scrambling facial features [60], removing or masking a face feature [61], and diminishing the visibility of faces by adding visual noise [62,63]. Another possible interpretation is that the N170 amplitude increase for inverted faces might be a result of the recruitment of additional processing resources in object perceptual systems [64], a hypothesis that is supported by some fMRI evidence [40,41]. Finally, considering that isolated features, eyes in particular, evoke a larger N170 than a whole face, Bentin et al. [55] and Itier et al. [65] proposed that the increase in N170 amplitude to inverted faces might be due to the processing of the eye region, which would rather support the qualitative account.

In the present study, we recorded ERPs while participants viewed face and house images parametrically rotated away from upright orientation in order to determine whether the N170 FIE reflects a quantitative and/or a qualitative change in face processing mode. This experimental design extends the parametric approach used in previous behavioral studies [21,22,28] and represents a step further in documenting the electrical brain responses that reflect the processing mechanisms that are allegedly occurring. This design also overcomes the limitations of previous ERP investigations often restricted to upright and inverted orientations by using intermediate levels of rotation [59,62]. Jacques and Rossion [59] also used a similar stimulus manipulation as in our study. Nonetheless, their study's goal was to relate P1 and N170 measures with participants' behavioral performances in a face-matching task, while the main purpose of the present study was to characterize the pattern of ERP responses to different orientations, and compare these results to those of house images. Our guiding hypotheses were the following: if FIE reflects a qualitative shift in processing mode (i.e., *qualitative hypothesis*), then changes in amplitude and latency of face-sensitive ERP components would exhibit a *discontinuity *function as faces were rotated away from upright orientation. On the contrary, if FIE results from a general difficulty processing configural and/or featural facial information (*quantitative hypothesis*) one would expect a rather *linear *increase in the amplitude and latency of these components with face rotation.

However, one cannot unequivocally disentangle the quantitative/qualitative accounts based only on the linear/nonlinear pattern of ERP changes with face rotation. Indeed, a nonlinear effect of rotation may simply suggest that the involved process(es) operate non-linearly rather than reflecting differences in processing mode. Therefore, to put tighter constraints on the qualitative/quantitative hypotheses listed above, we performed topographical and dipole source analyses, which will provide some insights about the neuro-anatomical loci of face rotation effects on scalp ERPs. Accordingly, if a discontinuity in face rotation functions reflects a qualitative difference in processing mode, one can expect to find topographical ERP changes of the ERP components sensitive to face rotation effects, reflecting the involvement of different neural sources between the two face orientations. Alternatively, the quantitative hypothesis would predict a complete spatial overlap of the neural sources involved in processing upright and inverted faces, the activity of which is expected to show an incremental increase as face orientation departs from upright position.

## Results

### Behavioral Results

**Figure 1:**
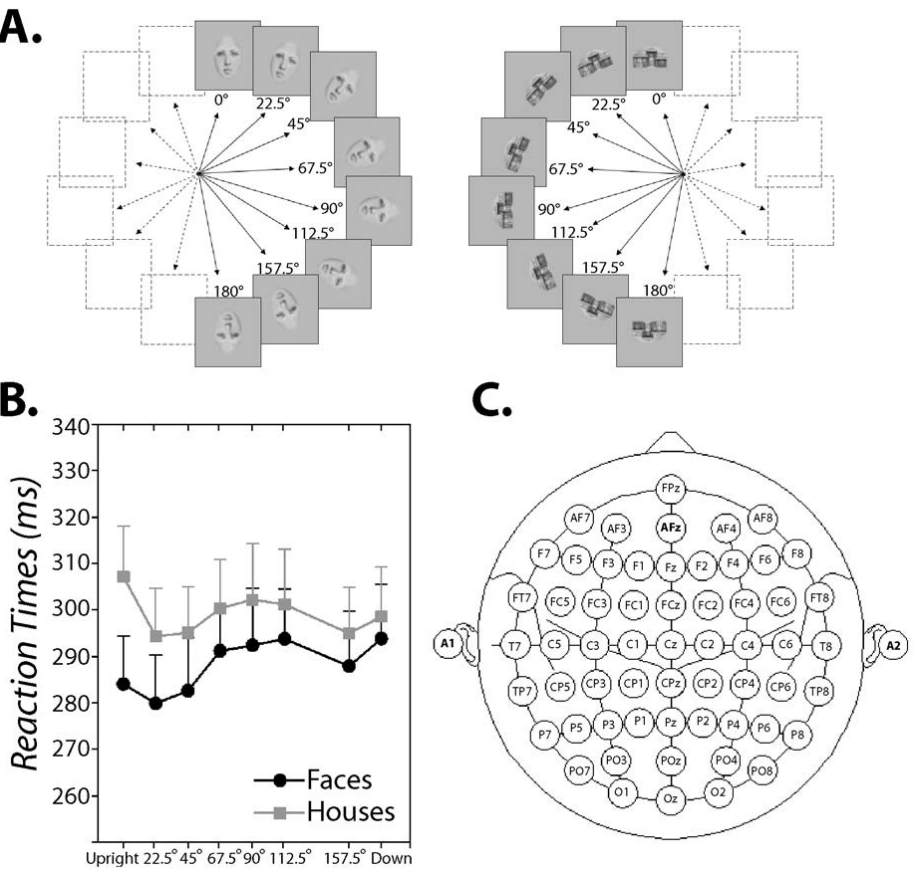
**Stimuli used, EEG layout and RT results**. **A**. A sample of face and house images used in this study. Face and house images were presented at eight angles of rotation (clockwise and counter-clockwise) one at a time in a random order. **B**. RTs of correct face (full black line) and house decisions (dashed black line) were plotted as a function of stimulus orientation. Error bars show one standard error of mean (SEM). **C**. Electrode layout for EEG recording.

Participants' accuracy in categorizing misrotated face and house images (see Figure 1A for stimuli illustration) was nearly perfect, averaging at 94.1% (*SD *= 1.4) and 94.5% (*SD *= 1.4) correct decisions respectively for face and house images. Image rotation did not affect participants' accuracy rates for both face and house images (p > .6). Mean reaction times (RTs) for correct responses to face and house images are shown in Figure 1B at each of the eight rotations. Overall, participants responded 20 ms faster to face than to house images (F(1, 14) = 10.03, p < .007). The main effect of rotation was significant (F(7, 98) = 3.03, p < .02, *ε *= .64), but the interaction between stimulus category and rotation did not reach significance (p > .06), indicating that the speed of both face and object decisions were similarly affected by image rotation. To better qualify the shape of RT curve displayed in Figure 1B, we examined the results of ANOVAs' polynomial contrasts. There was no evidence of a linear increase in RT as a function of angle of rotation (p > .1). Instead, the curvilinear shape of RT curve included two to three inflection points indicating the presence of cubic (F(1, 14) = 5.85, p < .03) and quartic (F(1, 14) = 9.24, p < .009) trends in RT data.

### ERP Results

The primary objectives of our study are: (1) identify the time moment at which rotation of face images affects electrical brain activity, (2) identify the pattern of electrical activity changes as a function of image rotation, in which case significant effects of rotation were supplemented with trend analyses using polynomial contrasts (3) and finally examine whether rotation effect would cause topographical distribution changes of the ERPs. To achieve this, both scalp current density (SCD) mapping and dipole source modeling were performed on scalp recorded ERP data of interest.

#### Effect of parametric rotation on ERP components (P1, N170 and VPP)

**Figure 2:**
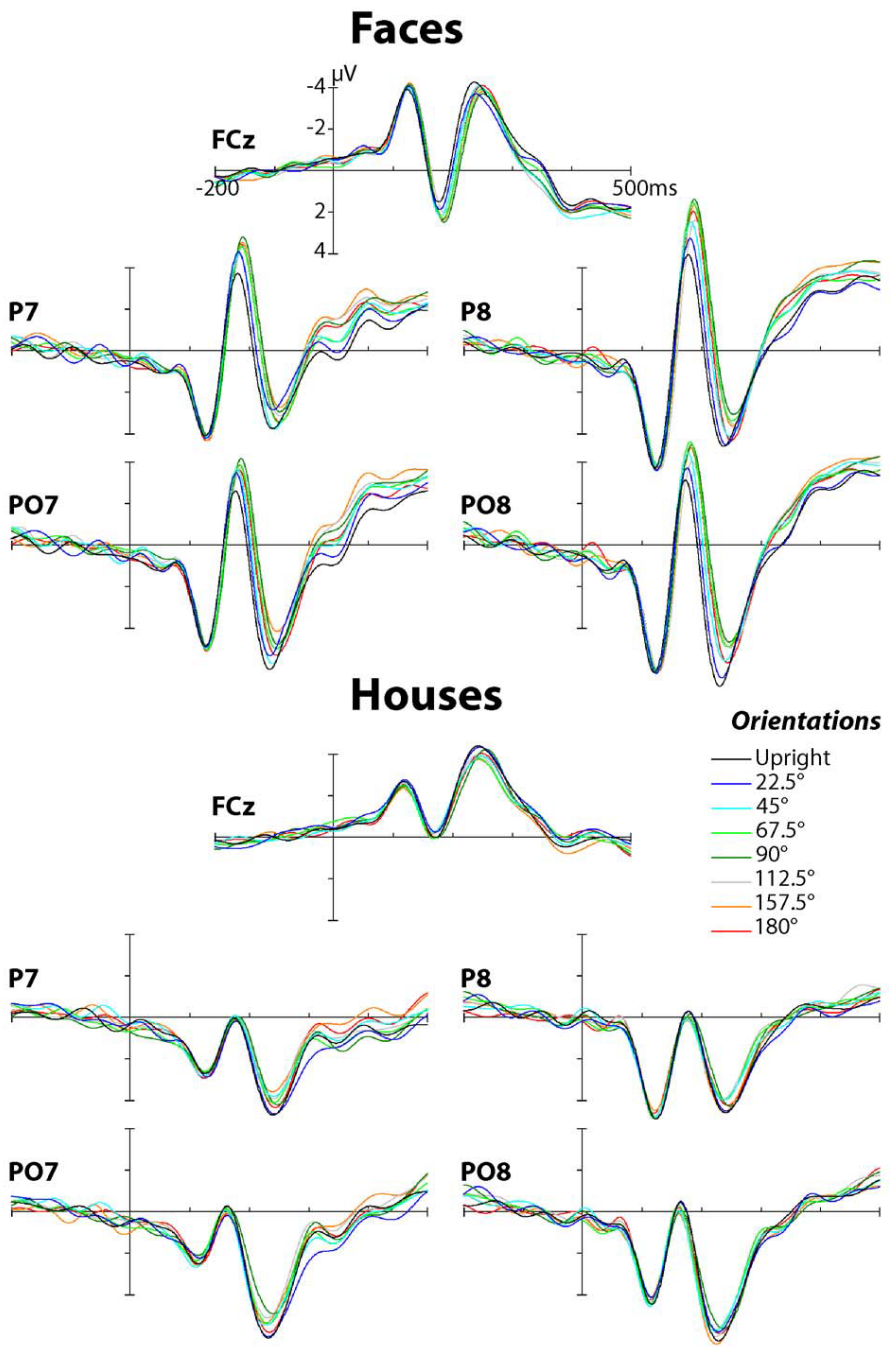
**Effect of picture plane rotation on early ERPs to face and house images**. Grand-average ERPs evoked by face (top panel) and house stimuli (bottom panel) overplotted for all eight angles of rotation. The depicted data are shown from lateral infero-temporal (PO7/PO8), occipito-temporal (P7/P8) scalp sites and from midline fronto-central site (FCz) with the peaks measured indicated.

**Figure 3:**
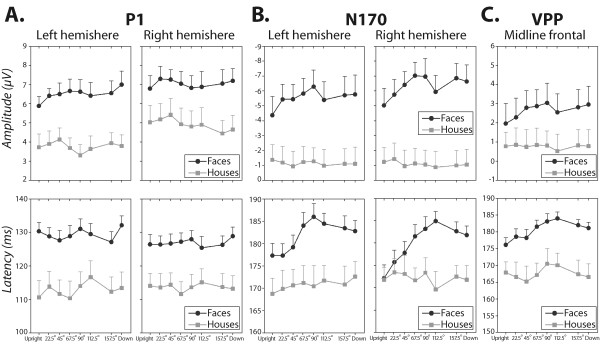
**Plots of P1, N170 and VPP measures for faces and houses as a function of rotation angles**. Amplitude (upper panel) and latency (lower panel) of P1 (**A**.), N170 (**B**.) and VPP (**C**.) peak responses to face (black circle) and house (grey square) images are plotted against stimulus orientation. **A**. For P1 plots, each datum point represents the average values of P1 measurements recorded over left (O1/PO7) and right (O2/PO8) occipital and infero-temporal scalp sites. **B**. For N170 plots, each datum point represents the average values of N170 measurements recorded over left (P7/PO7) and right (P8/PO8) occipito-temporal and infero-temporal scalp sites. **C**. For the VPP plots, each datum point represents the average values of VPP measurements recorded over midline frontal (Fz) and fronto-central (Fcz) scalp sites. Data plotted are means (*n *= 15) and vertical ranges represent + 1 SEM.

**Figure 4:**
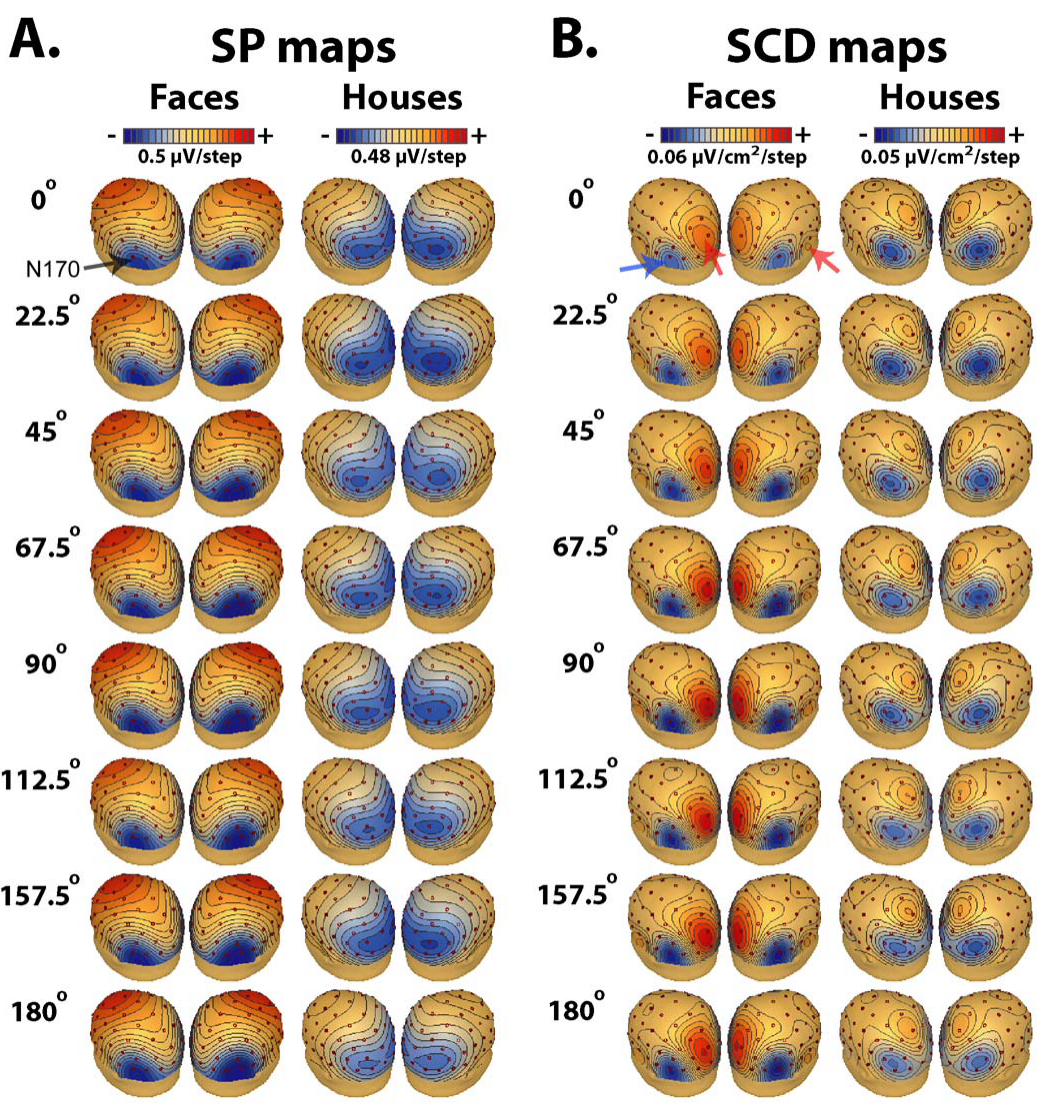
**Topographical SP and SCD maps of the N170 to face and house images at each angle of orientation**. **(A.) **Scalp Potential (SP) and **(B.) **Scalp Current Density (SCD) maps, arranged as 3 dimensional images as viewed from the left posterior top, illustrate SP and SCD topographical distribution of the N170 at its peak maximum for face (first column) and house (second column) images at each angle of orientation. Color grading of SCD maps shows areas with current sinks (negative) indicated with the red arrows, and areas with current sources (positive) with the blue arrow.

The grand mean ERP waveforms for face and house images presented at different angles of rotation are plotted in Figure 2 at selected scalp sites. The ERP waveforms for face and house images enclosed a positive-going deflection (P1) with a maximal peak amplitude at posterior scalp locations (O1/O2 and PO7/PO8) followed by a negative deflection (N170) which was prominent over occipito-temporal and infero-temporal scalp locations (P7/P8 and PO7/PO8). In the same latency range as the occipito-temporal N170, a vertex positive peak (VPP) was recorded over midline anterior scalp sites (Fz, FCz) for both stimulus categories. As shown in Figure 2, N170 and VPP peaks evoked by face images were strongly affected by image rotation (see also Figures 3B and 3C). There was no evidence for image rotation effect on P1 evoked by faces and houses (see also Figure 3A). The scalp potential (SP) and scalp current density (SCD) maps of the N170 evoked by face and house images at each image orientation are displayed in Figures 4A and 4B.

##### P1

A 2 (stimulus category) × 8 (angles of rotation) × 2 (electrodes) × 2 (laterality) statistical repeated-measures ANOVA performed on P1 amplitude and latency revealed a significant main effect of stimulus category, with faces eliciting larger and later P1 peaks than houses (F(1,14) > 28.46, p < .00015). There were no significant effects of rotation (F(7, 98) < 1.27, p > .2, *ε *= .51) and no significant interactions involving rotation and any other factor (all p > .2). As can be seen in Figure 3A, the amplitude and latency of P1 remained stable as the orientation of face and house images departed from upright position. The absence of rotation effect on P1 amplitude and latency measures also holds when contrasting upright and inverted images of faces and houses only (all p > .4).

##### N170

A 2 (stimulus category) × 8 (angles of rotation) × 2 (electrodes) × 2 (laterality) statistical repeated-measures ANOVA showed a significant main effect of rotation (amplitude: F(7, 98) = 2.86, p < .035, *ε *= .57; latency: F(7, 98) = 8.78, p < .00001, *ε *= .59) and a significant stimulus category × rotation interaction (amplitude: F(7, 98) = 3.58, p < .017, *ε *= .48; latency: F(7, 98) = 8.49, p < .00001, *ε *= .63). As can be seen in Figure 3B, image rotation increased the amplitude and latency of the N170 when elicited by faces (all F > 4.6, p < .005) and not when elicited by objects (all F < .51, p > .7). Similar to P1, there was a stimulus category main effect indicating larger and later N170 peaks for faces than for houses (F(1,14) > 11.45, p < .0045).

To investigate the pattern of N170 amplitude and latency increases as faces were rotated away from upright orientation we conducted separate polynomial analyses. As illustrated in Figure 3B, the curve shape reflecting N170 amplitude enhancement with rotation angle is clearly curvilinear rather than simply linear. It depicts a slight linear increase from upright to 90° of angle of rotation (p < .045), followed by a dip in the curve between 90° and 112.5° indicating a slight decrease in N170 amplitude (p < .025). This amplitude decrease remained stable thereafter. Trend analyses revealed an effect of high order components, a quadratic and sixth order trends (F(1,14) > 4.9, p < .045) in addition to a significant linear trend (F(1, 14) = 10.31, p < .006). The linear and high order (quadratic, sixth order) trends accounted respectively for 40.7% and 44.5% of the total variance in the data. The curve shape reflecting N170 latency enhancement as face orientation departed from upright view depicted at least one point of inflexion between 90° and 112.5° angles of rotation (cf. Figure 3B), suggesting a curvilinear above a linear trend. N170 latency to face images increased gradually from upright orientation to 90°–112.5° of rotation (p < .027), reached its maximum at 112.5° and then remained relatively stable at 157° and 180°. Polynomial contrast analyses confirmed the presence of a significant linear trend (F(1, 14) = 66.1, p < .0001) in addition to quadratic (F(1, 14) = 61.28, p < .0001) and cubic (F(1, 14) = 13.82, p < .002) trend effects. The linear trend accounted for 66.1% of the total variance in the data while the quadratic and cubic trends accounted for 30.1% of the data. The results of trend analyses suggest the involvement of at least two mechanisms during the processing of rotated faces.

##### VPP

A 2 (stimulus category) × 8 (angles of rotation) × 2 (electrodes) statistical repeated-measures ANOVA was performed on VPP amplitude and latency measures. The main effect of rotation did not reach significance level for amplitude measures (F(7, 98) = 2.15, p = .082, *ε *= .61) but was significant for latency measures (F(7, 98) = 5.22, p < .001, *ε *= .55). Although the interaction between stimulus category and rotation was not significant for both VPP amplitude and latency measures (all F < 1.67, p > .1), *post-hoc *tests showed that rotation produced main effects on the amplitude and latency of VPP when elicited by face images (all F > 2.68, p < .046) but not when elicited by house images (all F < 1.52, p > .1).

As can be seen in Figure 3C, rotation effects on VPP elicited by faces are smaller than those observed on the N170. The curve shape reflecting VPP amplitude enhancement with image rotation depicts a slight linear increase. This was confirmed by trend analysis results that revealed a significant linear trend only (F(1, 14) = 5.46, p < .035) accounting for 51% of the total variance in the data. VPP latency enhancement as a function of face rotation depicted at least one point of inflexion between 90° and 112.5° angles of rotation (cf. Figure 3C), suggesting a curvilinear above a linear trend. Polynomial contrast analyses confirmed the presence of significant linear (F(1, 14) = 58.29, p < .0001) and quadratic trends (F(1,14) = 18.77, p < .001) that accounted respectively for 59.3% and 27.4% of the total variance in the data.

#### Effect of parametric rotation on N170 SCD measures

So far scalp potential (SP) results show that image rotation affected the N170/VPP responses evoked by face images only. In order to investigate whether this effect induced topographical changes at the N170 latency range, we computed scalp current density (SCD) maps from the spherical spline interpolation of the surface voltage recordings (according to Pernier et al. [66] as implemented in the BESA program [67]). Presentation of the data as SCD maps served to enhance the contribution on the scalp of shallow cortical generators compared to deeper ones, which may provide some insights on the number and location of underlying cortical sources. Figures 4A and 4B depict respectively SP and SCD distributions at the moment of the N170 peak across image orientation for faces and houses.

**Figure 5:**
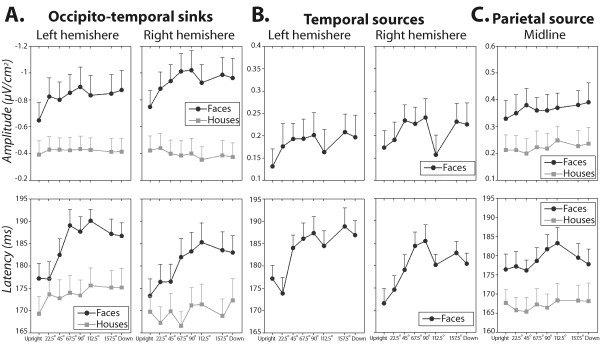
**Plots of SCD measures for faces and houses as a function of rotation angles**. Amplitude (upper panel) and latency (lower panel) of bilateral occipito-temporal current sinks (**A**.), bilateral temporal (**B**.) and midline parietal sources (**C**.) for face (black circle) and house (grey square) images are plotted against stimulus orientation. **A**. Occipito-temporal current sinks were measured over left and right occipito-temporal (P7/P8) scalp sites for face stimuli and over left and right parietal (P5/P6) scalp sites for houses. **B**. Temporal current sources were measured over left and right temporal scalp sites (T7/T8) for faces only. This current source activity pattern was absent in the SCD maps of houses. **C**. Parietal current sources were measured over midline parietal scalp site (Pz) for face and house stimuli. Data plotted are means (*n *= 15) and vertical ranges represent + 1 SEM.

The SCD maps for faces illustrate a bilateral current sink distributed respectively over left and right occipito-temporal (P7–P8) scalp sites with a concurrent but less pronounced bilateral positive current source over temporal (T7–T8) scalp sites. One-sample t-tests performed on the peak amplitudes of these temporal sources revealed that they differed significantly from zero at each face orientation condition (t(14) > 2.8, p < .014). In addition to this bilateral source/sink pattern, there was a strong positive focus (source) peaking slightly earlier over the midline parietal electrode site (Pz). As can be seen in Figure 4B, the fact that the distribution of the source/sink patterns identified in the SCD maps for faces showed little variations across image orientations indicate that at least two distinct brain regions contribute to the N170 evoked by faces. However their activity differed as a function of face rotations. Separate ANOVA analyses performed on the amplitude and latency of these source/sink SCD foci yielded a significant main effect of rotation on both peak amplitude and latency of the bilateral occipito-temporal sinks (F(7, 98) > 6.81, p < .001). The effect of rotation was significant for the latency of the concurrent temporal sources (F(7, 98) = 8.08, p < .0001, *ε *= .59) but was marginally significant for its amplitude (F(7, 98) = 2.41, p = .057, *ε *= .59). Only the latency of the parietal source was significantly modulated by image rotation (F(7, 98) = 3.89, p < .008, *ε *= .55). The curve shape reflecting amplitude and latency changes of the occipito-temporal/temporal sink and source complex with rotation was remarkably analogous to the pattern described for the N170 SP peak. For amplitude measures, a slight linear increase could be seen from upright to 90° rotation, especially over the right hemisphere (cf. Figures 5A &5B) followed by a dip in the curve at 112.5° rotation. For latency measures, the curves depicted at least one point of inflexion between 90° and 112.5° angles of rotation.

SCD maps for houses enclosed bilateral negative sinks which distribution was slightly shifted upward (P5 and P6) with respect to faces. There were however no concurrent temporal sources (t(14) < 1.34, p > .2) as identified in the SCD maps for faces. Besides these bilateral sinks, a positive current source can be seen over the midline parietal electrode site (Pz). As illustrated in Figures 5A and 5C, image rotation of houses had little effect on amplitude and latency measures of current sink (all F < 2.36, p > .08) and source activity (all F < 1.24, p > .2).

#### Dipolar source analyses

**Figure 6:**
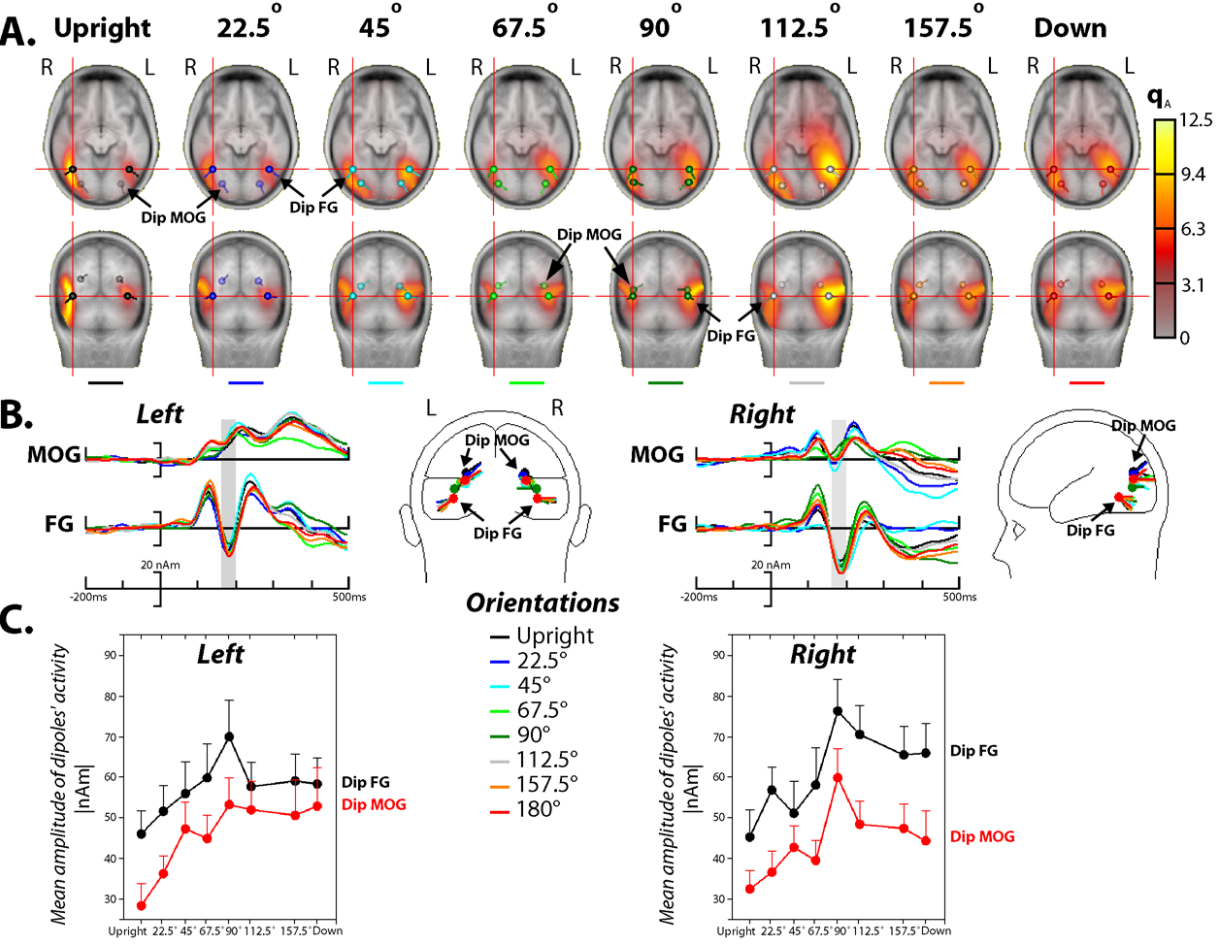
**Dipole source analyses of the N170 to faces performed at each angle of orientation**. **A**. Location of the two symmetrical dipoles (FG and MOG) and the computed MSPS result from the grand averaged ERP data for each face rotation are shown in an anatomical brain atlas space (the MNI averaged brain) on axial and coronal planes. The red color indicates the activity picked up by the MSPS. The activity is located near the FG and MOG dipoles, which suggests that the source model is adequate. **B**. Location of the two symmetrical FG and MOG sources from the grand averaged ERP data for each face rotation are displayed in a 4-shell spherical head model (BESA), with rear and lateral views, and their corresponding activity strength, expressed in units of nano-ampere-meters (nAm). **C**. The absolute values of the mean amplitude of the left and right FG and MOG source activity strengths are plotted against stimulus orientation. Data plotted are means (*n *= 15) and vertical ranges represent + 1 SEM.

Dipole (BESA) analyses were performed on the grand average ERPs elicited by face images at the eight angles of rotation and were restricted to the time-epoch of the N170. Source modeling was conducted in three stages. In stage 1, an initial solution was obtained by fitting both the location and the orientation of two symmetrical dipoles (one per hemisphere), which after several iterations from different start-up locations were found to be located in the fusiform gyrus FG, BA 37. Although the dipoles' location is consistent with neuroimaging data, its goodness-of-fit was highly variable across image rotation conditions. We thus added in the second stage two additional symmetrical dipoles, and then fit their location and orientation while constraining the location of the first dipole pair (FG dipoles). This procedure was applied to each ERP average obtained for each face orientation condition. The second dipole pair was located in the middle occipital gyrus MOG, BA 19, though its location in the x, y, and z axes varied slightly with angles of rotation, more notably for the 90° rotation (cf. Table 1 for dipoles' localization in the x, y, z Talairach coordinates and Figure 6). These two symmetrical dipoles explained on average more than 98.5% of the variance over the N170 time-epoch across all face rotation conditions (see Table 1 for time-intervals for the dipoles' fit and residual variance values). To examine how well the final model obtained for the grand-average ERPs fit individual data, the four-dipole model was applied to each individual N170 by only optimizing the orientation but not the location of the symmetrical FG and MOG dipoles. Table 1 shows that our final dipole solution was highly satisfactory for all subjects and for all face rotation conditions (the residual variances on average were below 6.24%).

**Table 1:** FG and MOG dipole sources of N170 responses to face images at each angle of image rotation.

								**Grand-average ERP**		**Individual ERP (*n *= 15)**	
*Orientations*		*FG Dipole*			*MOG Dipole*			*Time interval*	*%RV*	*Time interval*	*%RV (1 SEM)*
		*x*	*y*	*z*	*x*	*z*	*Y*				

Upright	Ta	± 39	-58	-7	± 28	-78	17	161–191 ms	1.47	155–185 ms	7.86 (1.5)
	MNI	± 40	-59	-12	± 28	-81	14				

22.5°	Tal	_	_	_	± 26	-78	15	164–194 ms	1.386	161–191 ms	6.95 (1.39)
	MNI				± 26	-81	12				

45°	Tal	_	_	_	± 28	-77	7	166–196 ms	1.448	166–196 ms	6.02 (1.46)
	MNI				± 28	-80	3				

67.5°	Tal	_	_	_	± 33	-78	8	169–199 ms	0.971	169–198 ms	6.8 (1.98)
	MNI				± 33	-81	4				

90°	Tal	_	_	_	± 38	-76	2	169–199 ms	1.139	172–202 ms	5.31 (1.03)
	MNI				± 38	-78	-2				

112.5°	Tal	_	_	_	± 28	-76	9	172–202 ms	0.941	172–202 ms	6.06 (1.67)
	MNI				± 28	-79	6				

157.5°	Tal	_	_	_	± 31	-78	9	172–202 ms	1.072	170–200 ms	5.02 (0.92)
	MNI				± 31	-81	6				

Down	Tal	_	_	_	± 29	-78	10	172–202 ms	1.034	169–199 ms	5.92 (1.18)
	MNI				± 29	-81	7				

Localization in the x, y, z Talairach (Tal) and MNI coordinates (mm) of left and right dipoles, i.e., Dipole 1 located in the fusiform gyrus (FG) and Dipole 2 in the middle occipital gyrus (MOG), the time-interval for the dipole fit and the resulting residual variance (% RV) for the grand-averaged and for the individual ERP data are displayed for each face rotation condition. For the individual ERP data, we report the average time-intervals used for the dipole fit and the statistical mean of residual variance (+ 1 SEM) obtained for each face rotation condition. Note that Dipole 1's location remained stable as we restrained its location across rotation conditions, while relaxing that of Dipole 2.

Figure 6 illustrates the location of the two symmetrical dipoles, the FG and MOG sources, obtained for each face rotation condition. In Figure 6A, the FG and MOG sources are displayed in the anatomical brain atlas space (the MNI averaged brain) and in Figure 6B in a 4-shell spherical head model (BESA) along with the dipoles' strength (source waveforms) obtained for the grand averaged data. The dipoles' strengths, especially those located over the right hemisphere, show reliable effects of rotation. This was statistically investigated by computing the mean amplitude of each dipole waveforms within a 30-ms time interval around the N170 peak in every single subject and for each face rotation condition. The left and right FG and MOG source activity strengths averaged over all subjects at each face rotation are plotted in Figure 6C. As can be seen in this figure, the effects of face rotation on dipoles' moment paralleled those obtained for the N170 amplitude measures, in that the dipoles' strength gradually increased as faces departed from upright and reached their maximum at 90° rotation. This was followed by a massive drop in the sources' activity at 112.5° and remained relatively stable thereafter. A main effect of rotation was found for the right FG source and for the left and right MOG sources (F(7, 98) > 4.47, p < .015). The pattern of these dipoles' strength changes was accounted for by both linear (p < .01) and high order trends (p < .04) suggesting that the activity of right FG and bilateral MOG sources reflected similar mechanisms that were affected similarly by face rotation. Moreover, the absence of face rotation effect on the left FG source activity (F(7, 98) = 1.99, p > .1, *ε *= .45) would indicate that even though this source is active during face perception, the mechanism that it underlies is not sensitive to face rotation.

## Discussion

The present results provide clear-cut evidence for the N170 response tuning to face orientation. The N170 ERP component evoked by faces was affected in a non-linear fashion by face rotation. By contrast, P1 was affected neither by face inversion nor by face rotation. For house images, both inversion and image rotation had no effect on early-evoked potentials (P1 and N170). These findings along with numerous other empirical data undoubtedly make a strong claim for the sensitivity of the N170 to face-specific perceptual processes (see also [59,62,63]). These and other behavioral and electrophysiological results will be discussed in the following with respect to competing accounts of FIE.

### Similar behavioral costs but different ERP effects of picture plane rotation of faces and houses

Our behavioral data revealed that picture plane rotation similarly affected response times to face and house images. The magnitude of image rotation effects on RT data reported here was quite smaller than that reported in previous studies using cognitively more challenging tasks [23,29-31,59]. Nonetheless, despite the simplicity of the participants' task (ie., a face-house categorization task), we found that increasingly, but moderately, longer response times were required to categorize increasingly misoriented images of faces and objects. Notably RT costs for rotated faces and houses were both nonlinear, revealing a dip in RT increase at 112.5° rotations which is consistent with previous reports [21-25,59,68-70]. Furthermore, contrary to RT data, only the early-evoked potentials to faces, namely the N170, mirrored the behavioral rotation effect. Neither P1 nor N170 elicited by house images showed amplitude and/or latency differences as houses were rotated away from the upright view. These findings thus indicate that rotation effect has a perceptual locus for faces and probably a post perceptual locus for objects. The perceptual locus account of FIE has been clearly stated within the encoding bottleneck hypothesis [11], according to which FIE and picture plane rotation effects disrupt the perceptual analysis of faces [7,11,13,22,71] rather than their memory representations [1].

Notwithstanding this, it is still unclear why unlike faces the encoding of houses was not taxed by picture plane rotation. Following some theoretical frameworks [9,45,72], one can speculate that since the perceptual processing of objects relies in a great extent on part-based processes, and that inversion is less detrimental for this kind of process, there should not be any impact of inversion on the perceptual analysis of objects. Another line of reasoning posits that inversion effect arises as a by-product of the default level of processing ("entry point") at which a given visual object is recognized [31]. While most objects are typically recognized at the basic level, recognition of other objects that benefited from extensive expertise (i.e., faces, words and letters) is naturally achieved at a more specific level of categorization [73]. It is now well-established that inversion effect can be observed for non-face objects for which participants were either experts [3] or had become experts after extensive training [74]. Inversion effect was also found to modulate the N170 to novel visual objects (i.e. Greebles) following expertise training [75] and to letter strings that are learned like faces over a lifetime of experience [76].

### Strong evidence for N170 sensitivity to perceptual mechanisms taking place during face perception

A compelling conclusion that is suggested by our findings is that the N170 (and its vertex polarity reversal, VPP) is the only ERP component sensitive to face inversion and thus should be intimately related to the perceptual mechanisms devoted to face processing [46]. Contrary to some previous studies showing FIE on P1/M1 [50,51,57], varying the angles of rotation of faces in our study substantially affected both the amplitude and latency of the N170 component but not P1. Moreover, although we found larger and more delayed P1 to faces than to houses, one ought to be cautious when directly comparing ERP responses to face and natural object stimuli that differ unavoidably in terms of their physical features [77]. Given that the face and house pictures used in our study and in many other studies were not equated in terms of frequency content, spectral energy, luminosity, etc., it is rather difficult to compare between categories without the risk of being misled by effects of low level visual properties. This methodological caveat, on the whole, does not undermine our main ERP findings showing differential rotation effects for faces and houses, and more importantly differential rotation effects on P1 and N170 to faces.

The functional dissociation between M/P1 and M/N170 in terms of low- and high-level visual processing of faces has been demonstrated by some previous studies. Jemel et al. [62] found that the N170 gradually emerged as the noise level diminished in face pictures while no such noise-induced modulations were observed at the level of P1. In Tanskanen & al.'s [63] study, MEG activity was recorded to faces masked by different spatial frequency noise (NSF) contents. The M1 response profile was driven by low level visual properties of the NSF presented either alone or to mask face images. Conversely, the M170 response was tied solely to the degree of visibility of faces. Finally, Jaques and Rossion [59] found that although P1 amplitude and latency measures were affected by face rotation, only the pattern of N170 modulation mirrored the pattern of the behavioral picture plane costs. In keeping with these findings, the present results show, if anything, the strong sensitivity of the N170 response to stimulus manipulations that exert tight constraints on the default mechanisms to process faces. It is worth noting however that, contrary to Jacques and Rossion findings, P1 component in our study was strikingly silent to picture plane rotation. There are different experimental factors that may have caused rotation effects on P1 in their study. In their experiment subjects had to indicate whether two sequentially presented faces portrayed the same individual or not, a task that may have placed higher demands on the attentional system across face rotations than the one we used here. Our more simpler face-house categorization task did not impose extra-cognitive demands, which explains the absence of rotation effects on P1 that is known to be highly sensitive to attentional factors. Nonetheless, despite this and other methodological differences, and although their report did not make any inferences regarding the functional significance of face rotation effects on ERPs, it generally supports our N170 findings and overarching proposal.

### Nonlinearity in N170 pattern changes with face rotations

The primary goal of our study was to gather some insights on whether N170 FIE reflects qualitative or quantitative processing differences as face orientation departs from the upright view. Our data show that the pattern of N170 response profile is consistent with the qualitative predictions, as the increase of both the amplitude and latency of the N170 component, and its concomitant positive counterpart, the VPP, showed a discontinuity somewhere between 90° and 112.5° rotations. N170 latency increased almost linearly for rotations further than 0° up to or slightly beyond the horizontal (90°); but then decreased slightly for further rotations up to 180°. For N170 amplitude measures, the curve qualifying the gradual increase of the N170 amplitude also exhibited a curvilinear falloff, with a dip at some point over the range of picture plane rotation (i.e., 112.5°). A similar pattern was observed for the amplitude and latency of the VPP component evoked by faces. This is partly consistent with an early study showing similar face rotation effects on the latency of the VPP component [52] and on the N170 as described in a more recent study [59]. Furthermore, it is worth noting that this pattern of orientation tuning of the N170 is not specific to faces. Comparable orientation effects on early ERP responses (i.e, N1) have recently been reported for alphanumeric characters [76], which like faces are commonly seen at a canonical upright view and for which perceptual expertise has been acquired through a lifetime experience.

The pattern of N170 orientation-sensitivity is also consistent with a set of data from cell-recording studies in macaques' superior temporal sulcus [78,79]. It is striking how closely the curvilinear effect of rotation on the ERP responses to faces reported so far resembles the curvilinear (roughly bell-shaped) pattern in orientation tuning of face-specific cells. Among the cells tested, Ashbridge et al. [78] found that 73% were selectively responsive to a specific orientation in the picture plane, particularly the upright view (68%). The gradual decrease in cells' firing rate as the image deviated from the upright view displayed a curvilinear pattern; at 45° or 90° away from a cell's preferred orientation firing was reduced by half and reached baseline at 135°. The orientation range through which occurred the dip in the function relating N170 responses and orientation is also in agreement with previous behavioral studies supporting the qualitative shift in face processing between 90° and 120° [21-25].

### Qualitative but also quantitative effects of face rotation: Insights from N170 topographical and source analyses

Trend analyses, topographical and source localization results however cast some doubt on whether our N170 findings strictly fit the qualitative account. Given that we also found significant linear trends accounting for the pattern of N170 amplitude and latency increase, this raises the possibility that quantitative changes might have also occurred [28]. An alternative model for our findings is that configural processing of faces occurs up to 90°–112.5° rotations, but that this processing becomes more difficult with increasing rotation angle, as reflected by the linear trends in the pre-discontinuity portion of the curve. Subsequently, at rotations greater than 112.5°, there are two possible explanations for the concurrent fall-off of the N170 results. One interpretation is that there is a qualitative shift in processing at this point, from configural to featural, as suggested by the qualitative account. An alternative interpretation is that configural processing persists for all rotations, and faces closer to being fully-inverted are merely easier to process than at 90°–112.5° rotations. According to this explanation, faces at 90° rotation present the greatest challenge to the configural processing system, rather than fully-inverted faces which have been assumed to be the most difficult to perceive in the past. In fact, it is likely that the configuration of a face at 90° rotation is even more severely disturbed than at 180°; i.e., the features in inverted faces are still one above the other but in the reverse order, whereas in 90° the features are one next to each other. Alternatively, it is also possible that configural and featural processes may be involved across the whole range of face orientations, but would operate in a non-linear fashion. Indeed, previous studies have provided evidence that configural/holistic information is not completely lost when faces are inverted, and that featural information is also used to process upright faces. These findings suggest that the discontinuity in the function of behavioral and ERP responses to misrotated faces does not necessarily imply that it is indicative of a qualitative change but rather non-linear variations in the efficiency of processing mode.

Topographical analyses conducted on scalp current density measures also suggest that the qualitative hypothesis cannot fully account for all reported results. More specifically, although SCD measures showed similar trends of misrotation effects to those observed for SP N170s, the distribution of current foci did not vary across rotations, a result that would likely indicate a spatial overlap between the brain sources activated for upright and misrotated faces. This contradicts the kind of neurofunctional predictions that could be inferred from the qualitative view. If a discontinuity in face rotation functions is likely to signal a shift from a configural to a piecemeal processing mode, one can expect the involvement of distinct brain regions as face orientation reached the critical 90°–112.5° rotation range. Although some fMRI findings are consistent with these predictions [40,41,44], no additional regions were found active during perception of inverted faces in other studies [6,36-39]. Instead, it was demonstrated that upright and inverted faces recruit similar overlapping brain networks albeit with a reduced activity for inverted faces. Some authors concluded that activity modulation of face selective regions (i.e. FFA) due to face inversion ultimately reflects the orientation tuning of these regions to upright views of faces rather than a qualitative difference in processing mechanism [6]. In a similar vein, a recent study showed that face-selective regions, the middle FG and inferior occipital gyrus, engage in both part-based and holistic processing [80] at a similar extent for upright and inverted faces [38] with some form of right hemisphere advantage for configural/holistic processing and left hemisphere advantage for part-based processing (cf. [38,81]).

In our study, we also performed dipolar source modeling to tentatively address the question whether the non-linear modulation of the N170 by face rotation resulted from the activation of distinct neural sources as face orientation departed from the upright view or from the involvement of the same configuration of generators. Bearing in mind that intra-cortical source localization inferred from scalp recorded electrical brain activity does not entail a single mathematical solution, our model remains clearly speculative; it is an attempt to describe the brain regions that could be activated during the N170 time-range across face orientations. Nonetheless, we have good reasons to believe that the dipole solutions we found were highly consistent across participants' data. We found that scalp recorded N170 was better explained by two bilateral dipole pairs located in the FG and MOG in each participant. The location of the MOG sources varied slightly with the different face rotation conditions with maximum variations at 90° rotations within the same area. It is worth noting however that although parts of the FG have been reliably shown to be active during face perception in fMRI studies, there is little evidence that the MOG could be involved. Furthermore, a recent study by Chen and collaborators [36] reported activations of the MOG to faces along other face-selective brain regions. Interestingly, the MOG was sensitive to face inversion and particularly to face image symmetry, which suggests that this region could be implicated in the coding of some kind of configural information. Keeping in mind these sets of findings, we found that the activity of the right FG and bilateral MOG sources was non-linearly modulated by face rotation, with a clear peak of the dipoles' strength at 90°. However the small modulations of the left FG dipole's activity as a function of orientation did not reach significance level. These results suggest that left and right FG and MOG sources displayed differential activity in response to a wide range of rotated faces, though being all involved in processing upright but also misrotated faces. Further studies are needed to determine which face properties (i.e., configural/holistic and featural) these brain sources are sensitive to.

## Conclusion

In conclusion, our study adds to a growing body of evidence suggesting that the paradigmatic face inversion effect results from a qualitative shift in processing upright and inverted faces [22]. Although the qualitative explanation predicted the curvilinear shape of N170 modulations by face misrotations, we presented some evidence that other alternative explanations are also viable. Our topographical and source modeling findings indicate rather a spatial overlap between the brain sources activated for upright and misrotated faces, probably underlying similar mechanisms to process upright and inverted faces. Because our face-house categorization task does not specifically address which type of processing mechanism is taxed by face rotation, further experiments should test this issue by more closely linking the pattern of N170 response changes as a function of face rotation with performance on behavioral tasks isolating configural and featural processing. Lastly, the present results firmly establish the orientation tuning of the face-sensitive N170 to faces and strongly support the idea that the best electrophysiological candidate underlying face processing mechanisms is tied to the occipito-temporal N170 component [58].

## Methods

### Participants

Seventeen right-handed, neurologically and psychiatrically healthy volunteers (7 males and 10 females), recruited by advertisement at McGill University and Université de Montreal took part in this study. All subjects were paid for their participation and written informed consent was obtained from all subjects. The experimental protocol was accepted by the Ethics Committee Board of Riviere-des-Prairies Hospital. Due to technical artifacts, the EEG-data of two participants were excluded from data analyses. The age of the remainder fifteen subjects (nine were female) ranged from 19 to 34 years (mean: 24.95 years). All subjects had normal or corrected-to-normal vision.

### Materials

The experimental stimuli were constructed using gray scale images of sixty faces and sixty houses. Face stimuli were created using IQ Biometrix's FACES LE™ software, version 3.0 . FACES LE™ is a database of more than 3,800 facial features that can be combined to create images of both genders belonging to any race. Selected features were automatically blended together to produce a high-quality, photo-like composite images of 30 Caucasian female and 30 Caucasian male faces. The face images were then processed with Adobe Photoshop so as to have the same size and to be placed uniformly within the frame so that the fixation point fell on the bridge of the nose. House images were collected from various web catalogues, and edited so as to have the same size. All stimuli were on a gray background.

### Procedure

Each participant sat in a stationary comfortable seat in a light- and sound-attenuated testing room facing a 17-inch computer monitor 1 meter away, so that the face and house images covered 7.1 degrees of visual angle. All stimuli were presented centrally on a gray screen for 150 ms with an 1000 ms inter-stimulus interval. Each face and house image was shown randomly in eight orientations, from the upright (0 degrees) to the upside-down (180 degrees) orientation with six intermediate angles of rotation: 22.5, 45, 67.5, 90, 112.5, and 157.5 degrees (cf. [28]). Half of the face and house images were rotated clockwise, and half counterclockwise (Figures 1A). The 960-stimulus presentation sequence was broken into 4 blocks of 240 images each, and randomized with respect to stimulus category and angle of rotation. Participants were asked to indicate as quickly and accurately as possible by pressing one of two response buttons with the index of the right or left hand whether the presented image was a face or a house regardless of its orientation. Response hands were counterbalanced across participants. The face house categorization task used in the present study, despite being less cognitively demanding than a face-matching task, is expected to elicit a picture-plane inversion effect both behaviorally and electrophysiologically. Indeed, the majority of previous ERP studies showing a FIE on the N170 component used even simpler tasks, e.g. a detection task of a specific object category [47,48,55] or an orientation judgment task (upright vs. inverted) [5,49,51,54,64,65]. The experiment was programmed and run on a Pentium III/200 computer using E-Prime version 1.1 Psychology Software Tools.

### EEG Recording and ERP analyses

The electroencephalogram (EEG) was recorded with electrically shielded Ag/AgCl electrodes from 58 scalp locations of the enhanced 10–20 system (cf. Figure 1C) embedded in an elastic Easy cap [82]. Two bipolar electrodes placed above and below the dominant eye (vertical EOG) and at the outer canthus of each eye (horizontal EOG) were used to monitor eye movements. Electrode impedances were kept below 5 KOhm. Electrode AFz was used as ground and a left earlobe electrode as a reference for all scalp electrodes. The right earlobe was actively recorded as an additional reference channel. The EEG and EOG were recorded continuously with a band-pass from DC to 100 Hz at a1024 Hz sampling rate, and stored along with the trigger codes. The EEG signal was filtered using a digital band-pass filter (0.03–30 Hz), off line re-referenced to both earlobe electrodes and then using an average reference [83]. EEG segments with eye-blinks and other artifacts were automatically rejected if i) the standard deviation of the EOG channels within a 200 ms sliding window exceeds 40 μV and if ii) the standard deviation of any scalp electrode exceeds 20 μV. Eye blinks were then detected and corrected by subtracting from the EEG the PCA-transformed EOG components for each electrode, weighted according VEOG propagation factors (computed via linear regression). Artifact-free EEG segments time-locked to stimuli onsets were averaged from 200 ms before and 500 ms after stimulus onset separately according to stimulus category (face and house) and according to the eight angles of rotation. Only trials with correct responses were used for the averages. Averages comprised between 27 and 60 trials with a mean of 50 trials. Baselines were computed in the interval from 200 to 0 ms prior stimulus onset and subtracted before averaging.

Amplitude and latency measures of the scalp ERPs, P1, N170 and VPP components, were performed for each subject and for each angle of rotation of face and house images at scalp sites where activity was maximally recorded: P1 was measured between 80 and 140 ms bilaterally at occipital (O1/O2) and infero-temporal (PO7/PO8) electrode sites, N170 between 140 and 200 ms bilaterally at infero-temporal (PO7/PO8) and occipito-temporal (P7/P8) electrode sites, and VPP between 140 and 200 ms at anterior midline electrode sites (FZ and FCz). The time windows were chosen after visual inspection of each data set to ensure that the peak of the component would fall within that window for all subjects.

### Topographical and Dipolar Source Analyses

In order to examine potential changes in the scalp distribution of ERPs of interest (i.e., N170) as a function of image plane rotation, we computed 3D scalp voltage topography maps and corresponding scalp current density (SCD) maps using BESA 5.1 software (MEGIS Software GmbH, Gräfelfing, Germany). SCD waveforms and maps, expressed in μV/cm^2^, are obtained by computing second spatial derivatives (the Laplacian) of the scalp field potentials [66]. SCD distributions show the scalp foci where the current either emerges (sources) from the brain into the scalp or enters from the scalp into the brain (sinks) and thus provide a more differentiated topographic picture than scalp potential data. In addition, SCDs enhance the contribution on the scalp of shallow cortical generators compared to deeper sources [66]. For each subject and image orientation, Laplacian waveforms were derived from the surface spline-interpolated SCD data at each recorded scalp location. A 130–220 ms time-window was used to measure the maximum peak amplitude and latency of sink and source patterns identified on SCD maps for faces and houses. For faces we identified three SCD foci taking the form of a bilateral occipito-temporal current sink peaking over P7–P8 with a concurrent bilateral temporal current source peaking over T7–T8, and a positive current source over the midline parietal electrode site (Pz). For houses, SCD maps enclosed bilateral negative sinks peaking over P5 and P6 scalp sites and a positive current source over Pz.

We also investigated the sources of the electric N170 potentials recorded from the scalp. Source analysis of the grand-averaged N170 elicited by faces at each image rotation was performed using the multidipole model approach [67] implemented in the brain electric source analysis (BESA) software. A 20-ms time-window was defined for source analysis on the basis of the global field power (GFP) centered on the moment of N170 grand-average peaks. Goodness-of-fit was estimated in terms of residual variance (RV), i.e., the percentage of data that could not be explained by the model. RV thus represents a measure of the validity of the model solution. The validity of the source solution was also validated with the multiple source probe scan (MSPS) procedure as implemented in BESA. In this procedure, the brain is scanned with a regional probe source added to the current dipole solution. MSPS calculates a value q by comparing the P power of the probe source at r location in the marked time interval P_(r)_, with the mean probe source power in the reference baseline interval P_(ref)_. If the calculated MSPS images show maxima around the location of the modeled brain sources, this would indicate that the scalp recorded ERP data have been modeled adequately.

### Statistical analyses

Statistical analyses were conducted on both behavioral and electrophysiological data using a repeated measures analysis of variance (ANOVA) with appropriate Greenhouse-Geisser corrections. The F value, the probability level following correction (p), and the *ε *value are reported. Accuracy (% correct face and house categorization responses) and reaction time (RT) data were analyzed separately, with the factors being stimulus category (Face *vs*. House) and orientation (8 rotations). Electrophysiological measures including the peak amplitudes and latencies of P1, N70 and VPP components, were submitted separately to ANOVAs with stimulus category, rotation and ERP components' corresponding measurement sites as within-subject factors. Significant main effects and interactions involving the experimental factors of either stimulus category or Angles of rotation, or both, were submitted to additional F contrast tests. An alpha level of p ≤ .05 was required for statistical significance. Differences in performance and in ERP responses as a function of rotation were then characterized by performing trend analyses, which are often used to evaluate the separate contributions of linear and nonlinear components using polynomial contrasts [84,85]. The aim of such a procedure is to indicate the general form of relationships between changes in the dependent variable to changes in the ordered independent variable. The use of this procedure here would help to pinpoint the trend of behavioral and electrical brain activity changes over an ordered independent variable, and to verify whether the effect of orientation could best be represented by a linear function or necessitates a higher-degree function (quadratic, cubic or quartic). A linear trend is characterized by the absence of any inflexion point in the function (i.e. a straight line) and would involve a theory in which there is a single process changing at a constant rate. A quadratic trend is characterized by the presence of a single inflexion point. The quartic and cubic trends are more complex and are described by the presence of two and three inflexion points respectively.

## Authors' contributions

BJ and SR designed the study. CL, JC and SR recruited participants, collected the EEG data and helped in data analyses. BJ conducted the analyses and wrote the manuscript. All authors read and approved the final manuscript.

## References

[B1] Valentine T (1988). Upside-down faces: A review of the effects of inversion upon face recognition. Br J Psychol.

[B2] Yin R (1969). Looking at upside-down faces. J Exp Psychol Gen.

[B3] Diamond R, Carey S (1986). Why faces are and are not special: an effect of expertise. J Exp Psychol Gen.

[B4] Rhodes G, Brake S, Atkinson AP (1993). What's lost in inverted faces?. Cognition.

[B5] Rossion B, Gauthier I, Tarr MJ, Despland P, Bruyer R, Linotte S, Crommelinck M (2000). The N170 occipito-temporal component is delayed and enhanced to inverted faces but not to inverted objects: An electrophysiological account of face-specific processes in the human brain. Neuroreport.

[B6] Yovel G, Kanwisher N (2005). The neural basis of the behavioral face-inversion effect. Cur Biol.

[B7] Bartlett JC, Searcy J (1993). Inversion and configuration of faces. Cogn Psychol.

[B8] Carey S, Diamond R (1977). From piecemeal to configurational representation of faces. Science.

[B9] Farah MJ, Tanaka JW, Drain HM (1995). What causes the face inversion effect?. J Exp Psychol Hum Percept Perform.

[B10] Farah MJ, Wilson AFW, Drain M, Tanaka JN (1998). What is "special" about face perception?. Psychol Rev.

[B11] Freire A, Lee K, Symons LA (2000). The face-inversion effect as a deficit in the encoding of configural information: Direct evidence. Perception.

[B12] Leder H, Bruce V (2000). When inverted faces are recognized: The role of configural information in face recognition. Q J Exp Psychol.

[B13] Searcy JH, Barlett JC (1996). Inversion and processing of component and spatial-relational information in faces. J Exp Psychol Hum Percept Perform.

[B14] Tanaka JW, Sengco JA (1997). Features and their configuration in face recognition. Mem Cognit.

[B15] Bäuml K-H, Schnelzer M, Zimmer A (1997). The influence of inversion on the judgment of facial and non-facial attributes. Acta Psychol.

[B16] Nachson I, Shechory M (2002). Effect of inversion on the recognition of external and internal facial features. Acta Psychol.

[B17] Rakover SS, Teucher B (1997). Facial inversion effects: Parts and whole relationship. Percept Psychophys.

[B18] Sekuler AB, Gaspar CM, Gold J, Bennett PJ (2004). Inversion leads to quantitative, not qualitative, changes in face processing. Cur Biol.

[B19] Riesenhuber M, Jarudi I, Gilad S, Sinha P (2004). Face processing in humans is compatible with a simple shape-based model of vision. Proc Biol Sci.

[B20] Boutet I, Chaudhuri A (2001). Multistability of overlapped face stimuli is dependent upon orientation. Perception.

[B21] McKone E (2004). Isolating the special component of face recognition: Peripheral identification and a Mooney face. J Exp Psychol Learn Mem Cogn.

[B22] Murray JE, Yong E, Rhodes G (2000). Revisiting the perception of upside-down faces. Psychol Sci.

[B23] Rossion B, Boremanse A (2008). Nonlinear relationship between holistic processing of individual faces and picture-plane rotation: Evidence from the face composite illusion. J Vision.

[B24] Schwaninger A, Mast F (1999). Why is face recognition so orientation-sensitive? Psychophysical evidence for an integrative model. Perception.

[B25] Stürzel F, Spillmann L (2000). Thatcher illusion: Dependence on angle of rotation. Perception.

[B26] Donnelly N, Hadwin JA, Cave K, Stevenage S (2003). Perceptual dominance of oriented faces mirrors the distribution of orientation tunings in inferotemporal neurons. Cogn Brain Res.

[B27] Bruyer R, Galvez C, Prairial C (1993). Effect of disorientation of visual analysis, familiarity decision and semantic decision on faces. Br J Psychol.

[B28] Collishaw SM, Hole GJ (2002). Is there a linear or a nonlinear relationship between rotation and configural processing of faces?. Perception.

[B29] Lewis MB (2001). The lady's not for turning: Rotation of the Thatcher illusion. Perception.

[B30] Valentine T, Bruce V (1988). Mental rotation of faces. Mem Cognit.

[B31] Jolicoeur P (1985). The time to name disorientated natural objects. Mem Cognit.

[B32] Haxby JV, Hoffman EA, Gobbini MI (2000). The distributed human neural system for face perception. Trends Cogn Sci.

[B33] Kanwisher N, McDermott J, Chun MM (1997). The fusiform face area: A module in human extrastriate cortex specialized for face perception. J Neurosci.

[B34] Gauthier I, Tarr MJ, Moylan J, Skudlarski P, Gore JC, Anderson AW (2000). The fusiform "face area" is part of a network that processes faces at the individual level. J Cogn Neurosci.

[B35] Halgren E, Dale AM, Sereno MI, Tootell RBH, Marinkovic K, Rosen BR (1999). Location of human face-selective cortex with respect to retinotopic areas. Hum Brain Map.

[B36] Chen CC, Kao K-LC, Tyler CW (2007). Face configuration processing in the human brain: The role of symmetry. Cereb Cortex.

[B37] Passarotti AM, Smith J, DeLano M, Huang J (2007). Developmental differences in the neural bases of the face inversion effect show progressive tuning of face-selective regions to the upright orientation. Neuroimage.

[B38] Yovel G, Kanwisher N (2004). Face perception domain specific, not process specific. Neuron.

[B39] Leube DT, Yoon HW, Rapp A, Erb M, Grodd W, Bartels M, Kircher TT (2003). Brain regions sensitive to the face inversion effect: A functional magnetic resonance imaging study in humans. Neurosci Lett.

[B40] Aguirre GK, Singh R, D'Esposito M (1999). Stimulus inversion and the responses of face and object-sensitive cortical areas. Neuroreport.

[B41] Haxby JV, Ungerleider LG, Clark VP, Schouten JL, Hoffman EA, Martin A (1999). The effect of face inversion on activity in human neural systems for face and object perception. Neuron.

[B42] Kanwisher N, Tong F, Nakayama K (1998). The effect of face inversion on the human fusiform face area. Cognition.

[B43] Tong F, Nakayama K, Moscovitch M, Weinrib O, Kanwisher N (2000). Response properties of the human fusiform face area. Cogn Neuropsychol.

[B44] Epstein RA, Higgins JS, Parker W, Aguirre GK, Cooperman S (2006). Cortical correlates of face and scene inversion: A comparison. Neuropsychologia.

[B45] Moscovitch M, Behrmann M, Winocur G (1997). What is special about face recognition? Nineteen experiments on a person with visual object agnosia and dyslexia but normal face recognition. J Cogn Neurosci.

[B46] Bentin S, Allison T, Puce A, Perez E, McCarthy G (1996). Electrophysiological studies of face perception in humans. J Cogn Neurosci.

[B47] Eimer M (2000). Event-related brain potentials distinguish processing stages involved in face perception and recognition. Clin Neurophysiol.

[B48] Eimer M (2000). The face-specific N170 component reflects late stages in the structural encoding of faces. Neuroreport.

[B49] Goffaux V, Gauthier I, Rossion B (2003). Spatial scale contribution to early visual differences between face and object processing. Cogn Brain Res.

[B50] Itier RJ, Taylor MJ (2002). Inversion and contrast polarity reversal affect both encoding and recognition processes of unfamiliar faces: A repetition study using ERPs. Neuroimage.

[B51] Itier RJ, Taylor MJ (2004). N170 or N1? Spatiotemporal differences between object and face processing using ERPs. Cereb Cortex.

[B52] Jeffreys DA (1993). The influence of stimulus orientation on the vertex positive scalp potential evoked by faces. Exp Brain Res.

[B53] Jeffreys DA (1996). Evoked potential studies of face and object processing. Vis Cogn.

[B54] Rossion B, Joyce CA, Cottrell GW, Tarr MJ (2003). Early lateralization and orientation tuning for face, word, and object processing in the visual cortex. Neuroimage.

[B55] Sagiv N, Bentin S (2001). Structural encoding of human and schematic faces: Holistic and part-based processes. J Cogn Neurosci.

[B56] Bötzel K, Grüsser O-J (1989). Electric brain potentials evoked by pictures of faces and non-faces: A search for "face-specific" EEG-potentials. Exp Brain Res.

[B57] Linkenkaer-Hansen K, Palva JM, Sams M, Hietanen JK, Aronen HJ, Ilmoniemi RJ (1998). Face-selective processing in human extrastriate cortex around 120 ms after stimulus onset revealed by magneto- and electroencephalography. Neurosci Lett.

[B58] Rossion B, Jacques C (2008). Does physical interstimulus variance account for early electrophysiological face sensitive responses in the human brain? Ten lessons on the N170. Neuroimage.

[B59] Jacques C, Rossion B (2007). Early electrophysiological responses to multiple face orientations correlate with individual discrimination performance in humans. Neuroimage.

[B60] George N, Evans J, Fiori N, Davidoff J, Renault B (1996). Brain events related to normal and moderately scrambled faces. Cogn Brain Res.

[B61] Eimer M (1998). Does the face-specific N170 component reflect the activity of a specialized eye processor?. Neuroreport.

[B62] Jemel B, Schuller AM, Cheref-Khan Y, Goffaux V, Crommelinck M, Bruyer R (2003). Stepwise emergence of the face-sensitive N170 event-related potential component. Neuroreport.

[B63] Tanskanen T, Näsänen R, Montez T, Päällysaho J, Hari R (2005). Face recognition and cortical responses show similar sensitivity to noise spatial frequency. Cereb Cortex.

[B64] Rossion B, Delvenne JF, Devatisse D, Goffaux V, Bruyer R, Crommelinck M, Guérit JM (1999). Spatio-temporal localization of the face inversion effect: An event-related potentials study. Biol Psychol.

[B65] Itier RJ, Alain C, Sedore K, McIntosh AR (2007). Early face processing specificity: It's in the eyes!. J Cogn Neurosci.

[B66] Pernier J, Perrin F, Bertrand O (1988). Scalp current density fields: Concept and properties. Electroencephalogr Clin Neurophysiol.

[B67] Scherg M, von Cramon D (1985). Two bilateral sources of the late AEP as identified by a spatio-temporal dipole model. Electroencephalogr Clin Neurophysiol.

[B68] De Caro SA, Reeves A (2000). Rotating objects to determine orientation, not identity: Evidence from a backward-masking/dual-task procedure. Percept Psychophys.

[B69] Lawson R, Jolicoeur P (1998). The effects of plane rotation on the recognition of brief masked pictures of familiar objects. Mem Cognit.

[B70] Lawson R, Jolicoeur P (2003). Recognition thresholds for plane-rotated pictures of familiar objects. Acta Psychol.

[B71] Edmonds AJ, Lewis MB (2007). The effect of rotation on configural encoding in a face-matching task. Perception.

[B72] Tanaka JW, Farah MJ (1993). Parts and wholes in face recognition. Q J Exp Psychol.

[B73] Tarr MJ, Cheng YD (2003). Learning to see faces and objects. Trends Cogn Sci.

[B74] Gauthier I, Williams P, Tarr MJ, Tanaka J (1998). Training 'greeble' experts: A framework for studying expert object recognition processes. Vision Res.

[B75] Rossion B, Gauthier I, Goffaux V, Tarr MJ, Crommelinck M (2002). Expertise training with novel objects leads to left-lateralized facelike electrophysiological responses. Psychol Sci.

[B76] Milivojevic B, Corballis MC, Hamm JP (2008). Orientation sensitivity of the N1 evoked by letters and digits. J Vision.

[B77] Rousselet GA, Husk JS, Bennett PJ, Sekuler AB (2005). Spatial scaling factors explain eccentricity effects on face ERPs. J Vision.

[B78] Ashbridge E, Perrett DI, Oram MW, Jellema T (2000). Effect of image orientation and size on object recognition: Responses of single units in the macaque monkey temporal cortex. Cogn Neuropsychol.

[B79] Perrett DI, Oram MW, Ashbridge E (1998). Evidence accumulation in cell populations responsive to faces: An account of generalisation of recognition without mental transformations. Cognition.

[B80] Harris A, Aguirre GK (2008). The representation of parts and wholes in face-selective cortex. J Cogn Neurosci.

[B81] Rossion B, Dricot L, Devolder A, Bodart JM, Crommelinck M, De Gelder B, Zoontjes R (2000). Hemispheric asymmetries for whole-based and part-based face processing in the human fusiform gyrus. J Cogn Neurosci.

[B82] Sharbrough F, Chatrian GE, Lesser RP, Lüders H, Nuwer M, Picton TW (1991). American electroencephalographic society guidelines for standard electrode position nomenclature. J Clin Neurophysiol.

[B83] Nunez PL (1981). Electric fields of the brain.

[B84] Kirk RE (1968). Experimental design: Procedures for the behavioral sciences.

[B85] Maxwell SE, Delaney HD (1990). Designing experiments and analyzing data.

